# Modified Adenovirus Prime-Protein Boost Clade C HIV Vaccine Strategy Results in Reduced Viral DNA in Blood and Tissues Following Tier 2 SHIV Challenge

**DOI:** 10.3389/fimmu.2020.626464

**Published:** 2021-02-15

**Authors:** Delphine C. Malherbe, Lo Vang, Jason Mendy, Philip T. Barnette, David A. Spencer, Jason Reed, Bettie W. Kareko, D. Noah Sather, Shilpi Pandey, Constantinos K. Wibmer, Harlan Robins, Deborah H. Fuller, Byung Park, Samir K. Lakhashe, James M. Wilson, Michael K. Axthelm, Ruth M. Ruprecht, Penny L. Moore, Jonah B. Sacha, Ann J. Hessell, Jeff Alexander, Nancy L. Haigwood

**Affiliations:** ^1^Oregon National Primate Research Center, Oregon Health & Science University, Beaverton, OR, United States; ^2^Emergent BioSolutions, San Diego, CA, United States; ^3^Vaccine & Gene Therapy Institute, Oregon Health & Science University, Beaverton, OR, United States; ^4^Department of Pediatrics, University of Washington, Seattle, WA, United States; ^5^Center for Global Infectious Disease Research, Seattle Children’s Research Institute, Seattle, WA, United States; ^6^Centre for HIV and STIs, National Institute for Communicable Diseases, of the National Health Laboratory Service, Johannesburg, South Africa; ^7^Faculty of Health Sciences, University of the Witwatersrand, Johannesburg, South Africa; ^8^Public Health Sciences Division, Fred Hutchinson Cancer Research Center, Seattle, WA, United States; ^9^Department of Microbiology, University of Washington, Seattle, WA, United States; ^10^Department of Virology and Immunology, Southwest National Primate Research Center, San Antonio, TX, United States; ^11^Texas Biomedical Research Institute, San Antonio, TX, United States; ^12^Gene Therapy Program, Department of Medicine, Perelman School of Medicine, University of Pennsylvania, Philadelphia, PA, United States; ^13^Division of Medical Virology, Department of Pathology, Institute of Infectious Diseases and Molecular Medicine, University of Cape Town, Cape Town, South Africa; ^14^Centre for the AIDS Programme of Research in South Africa (CAPRISA), University of KwaZulu-Natal, Durban, South Africa; ^15^Molecular Microbiology and Immunology, School of Medicine, Oregon Health & Science University, Portland, OR, United States

**Keywords:** adenovirus (Ad) vector, DNA vaccine, NHP, mucosal challenge, ADCC, neutralizing abs, HIV, SHIV challenge

## Abstract

Designing immunogens and improving delivery methods eliciting protective immunity is a paramount goal of HIV vaccine development. A comparative vaccine challenge study was performed in rhesus macaques using clade C HIV Envelope (Env) and SIV Gag antigens. One group was vaccinated using co-immunization with DNA Gag and Env expression plasmids cloned from a single timepoint and trimeric Env gp140 glycoprotein from one of these clones (DNA+Protein). The other group was a prime-boost regimen composed of two replicating simian (SAd7) adenovirus-vectored vaccines expressing Gag and one Env clone from the same timepoint as the DNA+Protein group paired with the same Env gp140 trimer (SAd7+Protein). The env genes were isolated from a single pre-peak neutralization timepoint approximately 1 year post infection in CAP257, an individual with a high degree of neutralization breadth. Both DNA+Protein and SAd7+Protein vaccine strategies elicited significant Env-specific T cell responses, lesser Gag-specific responses, and moderate frequencies of Env-specific T_FH_ cells. Both vaccine modalities readily elicited systemic and mucosal Env-specific IgG but not IgA. There was a higher frequency and magnitude of ADCC activity in the SAd7+Protein than the DNA+Protein arm. All macaques developed moderate Tier 1 heterologous neutralizing antibodies, while neutralization of Tier 1B or Tier 2 viruses was sporadic and found primarily in macaques in the SAd7+Protein group. Neither vaccine approach provided significant protection from viral acquisition against repeated titered mucosal challenges with a heterologous Tier 2 clade C SHIV. However, lymphoid and gut tissues collected at necropsy showed that animals in both vaccine groups each had significantly lower copies of viral DNA in individual tissues compared to levels in controls. In the SAd7+Protein-vaccinated macaques, total and peak PBMC viral DNA were significantly lower compared with controls. Taken together, this heterologous Tier 2 SHIV challenge study shows that combination vaccination with SAd7+Protein was superior to combination DNA+Protein in reducing viral seeding in tissues in the absence of protection from infection, thus emphasizing the priming role of replication-competent SAd7 vector. Despite the absence of correlates of protection, because antibody responses were significantly higher in this vaccine group, we hypothesize that vaccine-elicited antibodies contribute to limiting tissue viral seeding.

## Introduction

Despite the progress achieved in the last 30 years, immunogen design and optimization of delivery methods that elicit protective immunity remain vital goals of HIV vaccine development. The need is particularly acute in sub-Saharan Africa, which bears the heaviest infection burden, where the dominant genotype is clade C. Many early vaccine efforts have focused on clade B viruses that are predominant in the USA, Europe, and Australia. The critical role for HIV Envelope (Env)-directed antibodies and neutralizing antibodies (NAbs) for protection from infection has been firmly established in passive antibody studies in nonhuman primates [reviewed in (1)] as well as in recent vaccine studies (2). Viral CD8+ T cells are also crucial to blunting the initial infection and appear to be effective in some cases in clearing infection (3). Effectiveness in blocking and controlling infection depends upon both the quality and the magnitude of the response, and many questions remain as to how to enhance vaccine-induced humoral and cellular immunity.

The HIV *env* genes used in this study were isolated from subject CAP257, a participant in the CAPRISA cohort who developed substantial neutralization breadth within 18 months of infection with a clade C HIV-1 virus (4, 5). This subject was of particular interest due to the rapidity of her bNAb development, suggesting that early arising viral variants were effective in generating this response. We cloned *envs* from multiple time points during the waves of broadening NAbs in the CAP257 subject (6) and characterized the neutralization sensitivity of these variants when expressed as pseudoviruses against a panel of bNAbs. These *env* genes were evaluated in different combinations for their immunogenicity in rabbits and macaques using a DNA+Protein co-immunization strategy. The most immunogenic vaccine strategy tested employed CAP257 *envs* isolated 54 weeks post infection (54wpi), resulting in strong Tier 1 and modest Tier 2 NAbs with only three immunizations, and responses were not improved either by priming with a week 7 T/F-like *env* or by boosting with 93wpi *envs*. Thus, we chose these 54wpi *envs* as vaccine antigens in the protection study.

The search for more effective vaccine delivery methods has also been an intense area of study. Recombinant proteins, plasmid DNA and diverse viral vectors among other vaccine platforms have been explored with various success (7). Over the years, our group has focused on two different approaches using envs isolated from HIV patients who developed neutralization breadth: [1] a DNA+Protein co-immunization strategy (8–10) and [2] replicating human adenovirus 4 (Ad4) or simian adenovirus 7 (SAd7) prime followed by a protein boost (11, 12). Co-administration of protein with either DNA or a NYVAC vector was recently shown to be effective in inducing early and potent V1V2 responses in a phase 1b trial (13). Using clade B envs, we showed that our DNA+Protein vaccine regimen was highly immunogenic in rhesus macaques (10), generating autologous Tier 2 NAbs and Env-specific T_FH_ cells. In addition, using a clade C Env, the SAd7 prime-protein boost approach provided 35% protection and tissue virus reduction against a heterologous Tier 1 mucosal SHIV challenge (12). The current study in rhesus macaques compared two vaccine strategies for immunogenicity and protection delivering SIV Gag p24 and the CAP257 54wpi HIV clade C env antigens either by SAd7 prime followed by a recombinant Env protein boost or by DNA+Protein co-administration. Both strategies elicited antigen-specific humoral and cellular immune responses, but neither vaccine approach provided significant protection from viral acquisition against repeated titered mucosal challenges with a heterologous Tier 2 SHIV. Nevertheless, both regimens significantly lowered the cell-associated viral DNA in multiple tissues compared to the control group, thus potentially dampening the infection and providing clues to the development of more effective vaccines in the future.

## Materials and Methods

### Ethics Statement

The study was carried out in accordance with the recommendations described in the Guide for the Care and Use of Laboratory Animals of the National Institutes of Health and the United States Department of Agriculture. All animal work was approved by the Oregon Health & Science University (OHSU) West Campus Institutional Animal Care and Use Committee. Animal facilities at the Oregon National Primate Research Center (ONPRC) are accredited by the American Association for Accreditation of Laboratory Animal Care. All efforts were made to minimize animal suffering and all procedures involving potential pain were performed with the appropriate anesthetic or analgesic. The number of animals used in this study was scientifically justified based on statistical analyses of virological and immunological outcomes.

### Animals

Thirty male *M. mulatta* (rhesus macaques) between 3.3 and 4.7 years of age were pair-housed at the Oregon National Primate Research Center (ONPRC) in Beaverton, OR. All animals were free of Cercopithecine herpesvirus 1, D-type simian retrovirus, simian T-lymphotropic virus type 1 and SIV infection at the start of the study. All macaques were negative for MHC class I alleles Mamu A*01, B*08 and B*17. The twenty animals included in the immunogenicity phase were also serotyped for SAd7 pre-existing immunity (neutralizing antibodies). All procedures were performed according to regulations and protocols approved by the OHSU West Campus Institutional Animal Care and Use Committee.

### SAd7 Immunogens

The SAd7 adenovirus vectors used in this study express either clade C HIV-1 Env CAP257 54wpi_D gp160 glycoprotein or SIVmac239 Gag p55 (SIVgag). Recombinant SAd7 viral vectors were generated by direct cloning of the CMV and transgene cassettes into the adenoviral plasmid using homing endonucleases I-CeuI and PI-SceI. Replication competent viruses were generated by transfecting SwaI-linearized recombinant SAd7 plasmid DNA into HEK293 cells using FuGene HD transfection reagent (Roche Applied Sciences). The SAd virus vector capacity for transgene *in vitro* expression was determined by western blot analysis for Env or SIVgag; and broadly neutralizing antibody (PGT121, NIH45-46, 10E8, and PGT145) binding to Env glycoproteins expressed on the surface of HEK293 cells following recombinant SAd7Env vector infection. Briefly, for protein expression as evaluated by Western Blot, HEK293 cells were infected with 5×10^8^ vp/ml of SAd7 Env gp160 recombinant vectors. Cells were harvested 48 h later and resuspended in RIPA buffer (Thermo Scientific) supplemented with protease inhibitors. Cell lysates samples were run on a 4-10% SDS-PAGE and transferred to nitrocellulose prior to probing with polyclonal N4000 rabbit IgG anti-Env (primary Ab, NH) and HRP-conjugated anti-rabbit IgG Ab (secondary Ab, Bethyl Laboratories) and development with SuperSignal West Femto blotting detection system (Thermo Scientific). To characterize Env protein cell-surface expression, SAd7Env-infected cells were removed from flasks using cell scrapers, washed with PBS, blocked with 10% goat serum, then aliquots added to 96-well microplates. The plates were centrifuged and cell pellets resuspended with 1 µg/ml broadly neutralizing antibody. The cells were centrifuged again, washed with PBS and incubated with 2 µg/ml PE-conjugated goat F(ab’)2 anti-human IgG secondary detection antibody (Abcam). The cells were pelleted by centrifugation, fixed, final pellets resuspended in PBS, and then analyzed by flow cytometry. Histogram shifts compared to secondary antibody alone staining represented bNAb recognition of surface expressed Env. SAd7 recombinant viruses were then expanded and purified to obtain enough viral particles for vaccination.

### Plasmid DNA Immunogens

The motif-optimized env nucleotide sequences were synthesized and cloned into the pEMC* expression vector as previously described (6). The generation of the codon-optimized V1R-SIV Gag plasmid was described by Egan et al. (14). Plasmid DNA was coated on gold bullets at a dose of 2 µg total DNA as described (15). To verify that the Env DNA bullets were functional, COS-7 cells were transfected with the DNA carried by the gold beads and assessed for Env protein expression (16). For the V1R-SIV Gag DNA bullets, plasmid DNA was eluted from the bullet and used to transfect 293T cells. Twenty-four and 48 h post transfection, 293T cells were lyzed and cell lysates were assessed by western blot for SIV Gag expression by 55-2F12 monoclonal antibody.

### Recombinant gp140 Protein Immunogens

54wpi_D CAP257 gp140 trimeric protein was generated and produced in 293F cells as previously described (16–18).

### Immunization and Challenge

Macaques were assigned to three experimental groups (3 x 10) which were balanced for MHC class I alleles, age and body weight. NHPs seropositive for SAd7 were only included in the DNA+Protein group. Two experimental groups were vaccinated and the third group was the naïve control group which did not receive any vaccination. In the SAd7+Protein vaccine group (n = 10), NHPs were inoculated intranasally [0.5 x 10^11^viral particles (vp)] with the MAD300 (Teleflex Medical) mucosal atomization device and intramuscularly (IM) (1x10^11^ vp) in the quadriceps with SIV gag and CAP257 54wpi_D env SAd7 vectors at weeks 0 and 4. They also received IM 50 µg CAP 257 gp140 Env protein boosts in the presence of Adjuplex adjuvant (Sigma) at weeks 4 and 12. The protein/adjuvant mixture was delivered into a different muscle group than the SAd7 vector combinations. NHPs in the DNA+Protein vaccine group (n = 10) were co-immunized with HIV clade C CAP257 54wpi_D Env gp140 protein; and with HIV clade C CAP257 54wpi_D env gp160 and SIVmac239 Gag as DNA plasmids at weeks 0 and 4. At week 12, NHPs in this vaccine group were co-immunized with HIV clade C CAP257 54wpi_D Env gp140 protein and three env DNA plasmids (CAP257 54wpi_D, 54wpi_C and 54wpi_G). The protein (50 µg) was delivered IM and the plasmids (36 µg) were delivered intradermally by PMED Gene Gun in 18 sites on the shaved abdomen and upper thighs as previously described (10). Regular blood draws were used to monitor immune responses in plasma and PBMCs during the immunogenicity phase. Inguinal lymph node biopsies were performed 3 weeks after the second and third immunizations. Two weeks after the second and third immunizations, buccal secretions were collected on Weck-Cel spears and extracted as previously described (19). One month after the last immunization, animals were exposed with up to 10 weekly IR (defined earlier) challenges with the Tier 2 clade C SHIV-1157ipd3N4 virus (5000 TCID_50_), as previously described (20, 21), until all controls (n = 10) were infected. Blood samples were obtained regularly to monitor immune responses in plasma and PBMCs during the challenge phase.

### ELISA Antibody Assays

HIV-1 Env binding antibody titers were measured in plasma samples collected at regular intervals against autologous CAP257 54wpi_D gp140 according to methods previously established (6). Buccal secretions collected 2 weeks after the second and third immunizations were assayed in an ELISA assay against autologous CAP257 54wpi_D gp140 Env protein as previously described (6).

### Neutralization Assay

The TZM-bl neutralization assay was performed as previously described (16) All values were calculated with respect to virus only wells [(RLU value for virus only minus cells only) minus (value for serum minus cells only)] divided by (value for virus minus cells only).

### ELISPOT Assay

IFN-γ ELISPOT was performed on fresh PBMCs as previously described (12, 22). Briefly, 1 x 10^5^ PBMC were incubated in duplicate overnight with 10 µM of the indicated Gag or clade C consensus Env 15-mer overlapping peptides. Results are IFN-γ spot-forming cells (SFCs) per 1 x 10^6^ PBMC following subtraction of duplicate wells with media only (negative control) and are considered positive if greater than twice the background and greater than 50 SFCs/1 x 10^6^ PBMC.

### Antibody-Dependent Cellular Cytotoxicity Assay

Using the assay developed by Alpert et al. (15), ADCC measurements were made with SHIV-SF162P3-infected target cells. Briefly, NKR24 cells were used as targets which were derived from CEM.NKR.CCR5 CD4+ T cells (23, 24) and obtained from the AIDS Research and Reference Reagent Program. The KHYG-1 rhCD16 effector cells were derived from the CD16-negative human NK cell line KHYG-1 (Japan Health Sciences Foundation) (25). Briefly, the NKR24 luciferase-reporter cell line is infected by spinoculation and incubated for 3–4 days before starting the assay to achieve an infection titer that is at least five-fold over background (Relative Light Units (RLU) readout of mock infected cells). SHIV-infected NKR24 target cells are washed and combined with KHYG-1 rhCD16 cells at a ratio of effectors to targets (E:T) of 10:1 and added to the assay plate along with serially diluted plasma or positive and negative mAb controls. Controls that define 100% RLU and 0% RLU are included on the plate. The assay is incubated for 8 h after which the luciferase substrate reagent, Bright-Glo (Promega), is used to read activity as RLU indicate luciferase activity which is an indicator of cytotoxicity of infected target cells. Data are reported as the %RLU and 50% ADCC titers (26).

### Follicular Helper CD4 T Cells Activation-Induced Marker Assay (AIM)

AIM is both a highly sensitive and specific assay to detect Ag-specific GC T_FH_ cells in rhesus macaques and is adapted here from the (AIM) assay (27). Briefly, lymphocytes were isolated from inguinal lymph node biopsies 3 weeks after the second and third immunizations. Purified lymphocytes (5 x 10^6^) were separated into three groups of 1x 10^6^ cells each: no exogenous stimulation, Ag stimulation 5 µg/ml CAP257 Env gp140 glycoprotein, 2 µg each SIVmac239 Gag ORF peptides (125 overlapping 15-mer peptides), or SEB (1 µg/ml) and incubated for 18 h at 37°C. Following stimulation, the cells were stained for 1 h: CD4 (L200), CD45RA (BV421, clone 59), OX40 (CD134, PE, clone 59), CD20 (APC-H7, clone 2H7), PD-1 (EH12.2H7), CD25 (BC96), CXCR5 (MU5UBEE). The frequency of antigen-specific CD25+OX40+ GC T_FH_ cells among total CD4+ T cells is determined by subtracting the frequency of CD25/OX40 cells in the “no exogenous” stimulation condition from the Env-antigen stimulation condition.

### Viral Loads

Quantitative PCR assays were performed on coded DNA samples (plasma, PBMC, or tissues) using methods described in detail previously (28).

### Statistical Analyses

Heterologous neutralization responses and ADCC AUC titers elicited by both vaccine strategies were assessed by Mann-Whitney test. For the ELISPOT and T_FH_ responses, repeated measures ANOVA with vaccine strategies (DNA/SAd7) and antigens (Env/Gag) as between group factors and time post challenge (weeks) as within group factor was used to compare response difference between groups and type in overall as well as at given time point. Due to the skewed distribution of ELISPOT and T_FH_ responses, the response values were transformed using logarithmic function with natural base. CS, compound symmetry, was chosen to be correlation within subject using BIC (Bayesian Information Criteria). Tukey-Kramer was used for multiple comparison correction. Due to the skewed distribution of viral loads, the viral load values were transformed using logarithmic function with natural base. For the longitudinal plasma and cell-associated viral loads, repeated measures ANOVA with vaccine strategies as between group factor and time post challenge (weeks) as within group factor was used to compare viral loads difference between groups in overall as well as at given time point. For the longitudinal plasma viral loads, AR (1), first auto-regressive, was chosen to be correlation within subject using BIC. Tukey-Kramer was used for multiple comparison correction. For the longitudinal cell associated viral loads, unstructured covariance was chosen to be correlation within subject using BIC. Tukey-Kramer was used for multiple comparison correction. For AUC viral loads, peak viral loads and for tissue viral loads, one-way ANOVA was used to compare difference between vaccine strategies and Tukey-Kramer was used for multiple comparison correction. The statistical analyses were performed either with GraphPad Prism, or SAS software packages. The Kaplan-Meier method was used to evaluate the effect of onset of infection, and log-rank test was used to compare distributions of time to infect between vaccine groups and control.

## Results

### Characterization of SAd7-CAP257 54wpi_D Immunogen and Study Design

**Figure 1:**
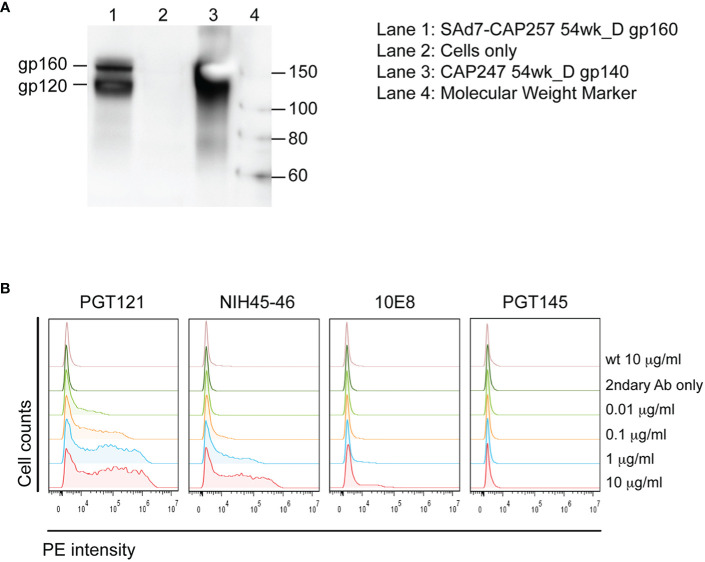
SAd7 expression of Env protein and antigenic characterization of SAd7-CAP257 Env. **(A)** Transgene expression was assessed by infecting HEK293 cells with SAd7 CAP257 54wpi_D Env gp160 virus and performing a western blot analysis of lysates collected 48 h post-infection. **(B)** Recognition of cell surface CAP257 54wpi_D Env by bNAbs targeting different epitopes was determined by flow cytometry. HEK293 cells were harvested 18 h after infection with SAd7 CAP257 54wpi_D Env gp160 and flow cytometry performed using increasing concentrations of four bNAb as primary and goat anti-human R-PE as secondary antibodies. Binding of the secondary antibody alone is indicated by “2ndary Ab only”.

A SAd7 vector expressing clade C HIV CAP257 54wpi_D gp160 Env glycoprotein was generated as described in *Materials and Methods*. The viral genome and transgene insertion cassette containing the motif-optimized 54wpi_D gp160 DNA were confirmed to be intact, the correct size, and without deletions or mutations by restriction digest, PCR and sequencing (data not shown). HIV Env protein expression was evaluated by western blot analysis after infection of HEK293 cells with the SAd7 vector, and correct expression products were detected as shown in **Figure 1A**. CAP257 54wpi_D Env glycoprotein expressed on the surface of SAd7-infected cells was recognized by Env-specific bNAbs (**Figure 1B**) that neutralized CAP257 54wpi_D pseudoviruses (5), thus demonstrating the presence of these epitopes on the SAd7-expressed Env. The selected CAP257 full-length envs were motif optimized for expression in mammalian cells, and plasmid DNA vaccines expressing Env gp160 were tested as pseudoviruses for neutralization by bNAbs. CAP257 54wpi_D Env protein trimer included as protein boosts was produced as recombinant gp140 and assessed for its antigenicity. Using biophysical methods to confirm antigenic conformation and epitope accessibility, 54wpi_D Env protein was tested for binding to well-defined broadly neutralizing antibodies (bNAbs) that have been shown to interact with the Envs expressed from clones isolated from subject CAP257 (5, 6).

**Figure 2:**
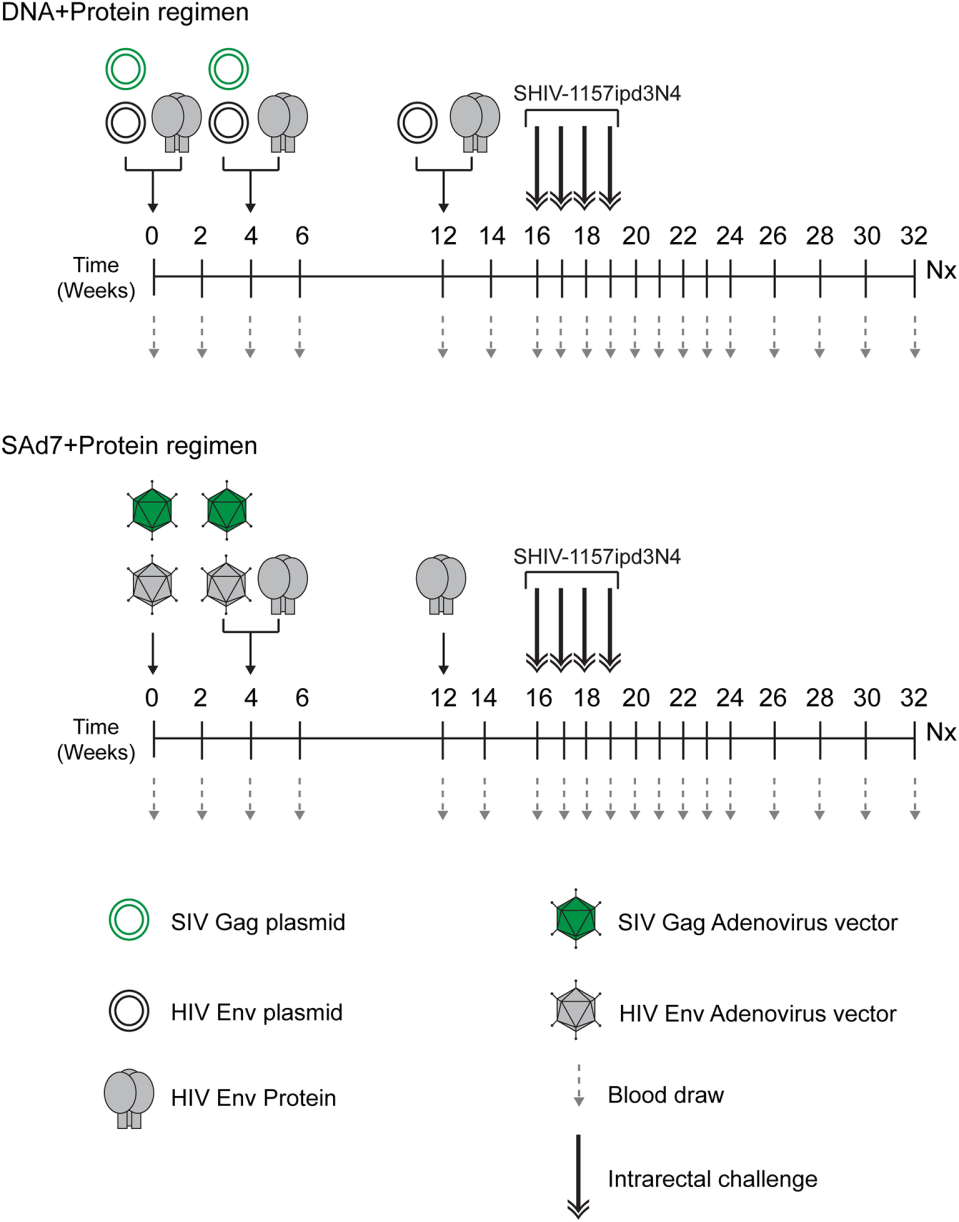
Vaccine/Challenge study design. NHPs in the DNA+Protein vaccine group (n = 10) were co-immunized with HIV clade C CAP257 54wpi_D Env gp140 protein; and with HIV clade C CAP257 54wpi_D env gp160 and SIVmac239 Gag as DNA plasmids at weeks 0 and 4. At week 12, NHPs in this vaccine group were co-immunized with HIV clade C CAP257 54wpi_D Env gp140 protein and three DNA plasmids (CAP257 54wpi_D, 54wpi_C and 54wpi_G). The protein (50 µg) was delivered IM and the plasmids (36 µg) were delivered intradermally by Gene Gun. NHPs in the SAd7+Protein vaccine groups (n = 10) were inoculated with HIV clade C CAP257 54wpi_D Env gp160 and SIVmac239 Gag as simian adenovirus 7 vectored-constructs intranasally (0.5 x 10^11^ vp/ml) and *via* IM (1 x 10^11^ vp/ml) at weeks 0 and 4. They also received IM CAP25754wpi_D Env gp140 protein boosts (50 µg) at weeks 4 and 12. After a month rest period, macaques were weekly challenged intrarectally with 5000 TCID_50_ Tier 2 clade C SHIV-1157ipd3N4 until all controls (n = 10) were infected.

The study (**Figure 2** and **Table 1**) consisted of two vaccine groups (n=10 each) receiving either SAd7+Protein or DNA+Protein vaccines and a challenge phase control group that was not vaccinated. Groups were negative for MHC class I alleles Mamu-A1*01, B*08, and B*17. Macaques were also screened for SAd7 seropositivity, and the seropositive animals were placed in the DNA+Protein group to avoid any anti-vector pre-existing immunity. In the SAd7+Protein group, macaques were primed IM and IN with SAd7 vector combinations 54wpi_D and SIV Gag at week 0, while at week 4, they received the same SAd7 vectors (IN and IM) and a protein boost IM. The SAd7 vector combinations were co-delivered IN and IM at 0.5x10^11^ vp *via* IN and 10^11^ vp *via* IM. The protein boost (50 µg) was delivered IM with Adjuplex adjuvant, in a different muscle group from the SAd7 vaccines. At week 12, animals received a final Env 54wpi_D gp140 protein boost IM.

**Table 1:** Vaccine components included in each strategy.

Strategy	Immunization #1		Immunization #2		Immunization #3	
	DNA^a^/SAd7^b^	Protein^c^	DNA^a^/SAd7^b^	Protein^c^	DNA^a^/SAd7^b^	Protein^c^
**DNA +** **Protein**	54wpi_D SIV Gag	54wpi_D	54wpi_D SIV Gag	54wpi_D	54wpi_D 54wpi_C 54wpi_G	54wpi_D
**SAd7 +** **Protein**	54wpi_D SIV Gag		54wpi_D SIV Gag	54wpi_D		54wpi_D

^a^DNA plasmid expresses Env gp160 heterotrimer, where each protomer is 160 kDa with an intact cleavage site.

^b^SAd7 vector expresses Env gp160 heterotrimer, where each protomer is 160 kDa with an intact cleavage site.

^c^HIV Envelope purified glycoprotein is a trimer, where each protomer is 140 kDa, no cleavage site.

Animals in the DNA+Protein vaccine group were co-immunized with HIV clade C CAP257 54wpi_D Env gp140 protein and with HIV clade C CAP257 54wpi_D env gp160 and SIVmac239 Gag as DNA plasmids at weeks 0 and 4. At week 12, macaques in this vaccine group were co-immunized with CAP257 54wpi_D Env gp140 protein and three DNA plasmids from the same timepoint expressing variants of Env (CAP257 54wpi_D, 54wpi_C, and 54wpi_G). The protein (50 µg) was delivered IM with Adjuplex adjuvant and the plasmids (36 µg) were delivered intradermally by PMED Gene Gun (29).

Four weeks after the last immunization, animals in all groups were challenged weekly intrarectally (IR) with 5000 TCID_50_ clade C Tier 2 SHIV-1157ipd3N4 (20) (21) until all controls (n = 10) were infected. Macaques were followed up for 16–18 weeks for virological outcomes and anamnestic immunity.

### Immunogen-Specific ELISPOT and T_FH_ Responses

**Figure 3:**
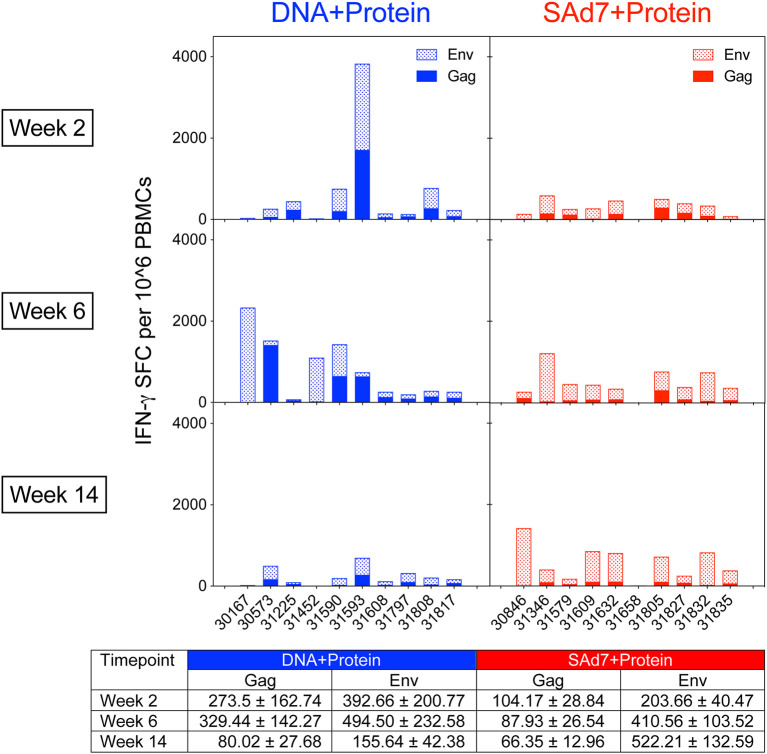
Env-specific and Gag-specific IFN-γ T cell responses. Two weeks after each immunization, macaque PBMCs were tested for IFN-γ production by ELISPOT after stimulation with pools of overlapping peptides for clade C consensus Env or SIVmac239 Gag. Data are expressed as background subtracted spot forming cell (SFC) per million PBMCs. Tabulated data show mean +/- SEM.

Cellular immune responses elicited by both vaccine strategies were assessed longitudinally. Env- and Gag-specific T cell responses were detected in macaque PBMCs in both vaccine groups as measured by IFN-γ ELISPOT assays (**Figure 3**) over the course of the study. The Env and Gag response patterns were both vaccine component- and animal-dependent. Overall, the Env responses were significantly greater than the Gag-specific responses (P < 0.0001) and significant within both vaccine groups (DNA group P = 0.0467; SAd7 group P = 0.0005). Responses to Env compared to Gag were greatest at week 14 after three immunizations (P = 0.0026), and the SAd7 group, but not the DNA group, maintained significantly higher Env responses through the third immunization (P = 0.0138).

**Figure 4:**
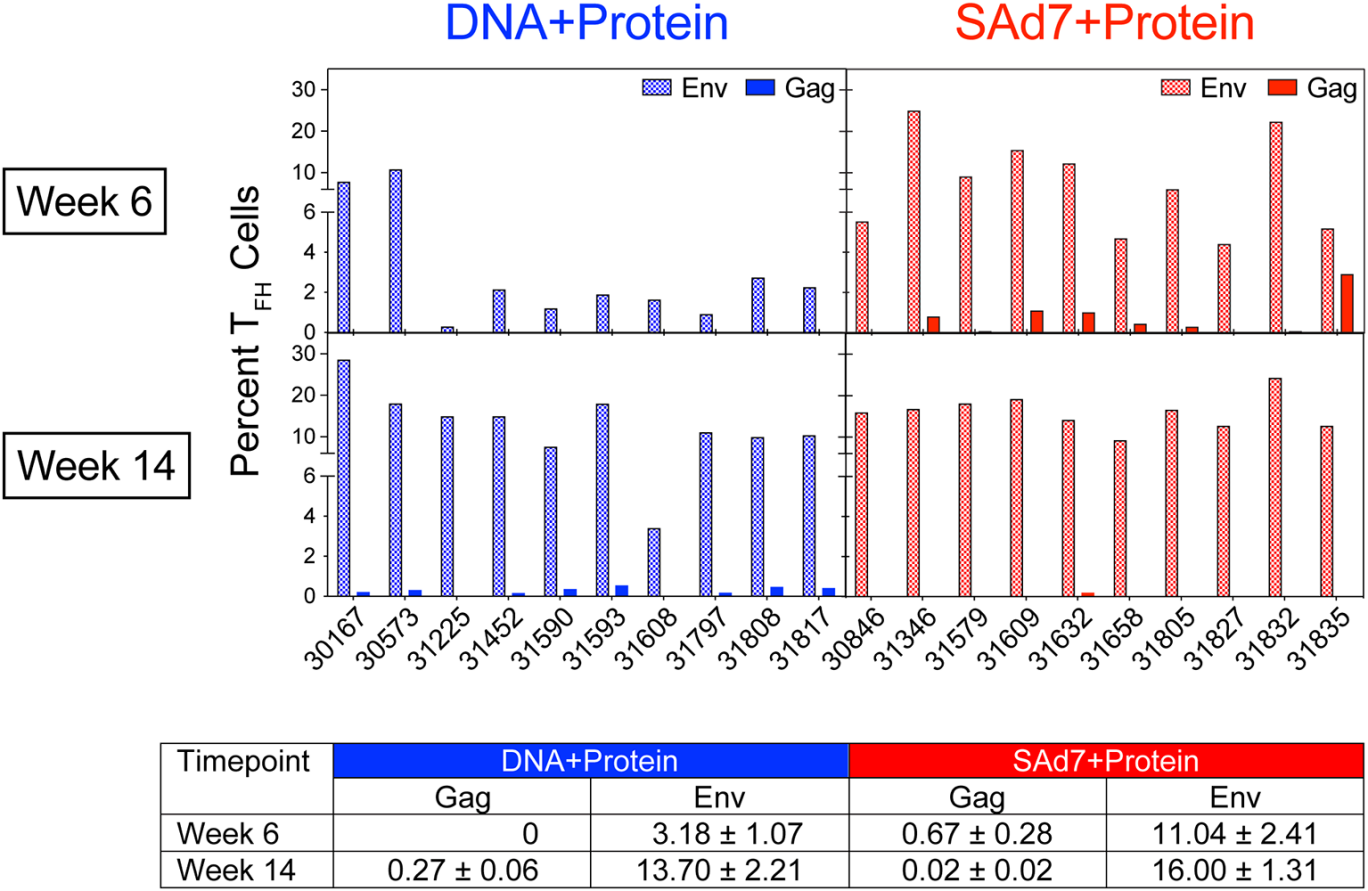
Env-specific and Gag-specific CD4 T_FH_ responses. Two weeks after the second and third immunizations, frequency of CD4 T_FH_ in inguinal lymph nodes was assessed after stimulation with CAP257 54wpi_D Env trimeric glycoprotein or a pool of 125 SIVmac239 Gag open reading frame (ORF) 15-mer overlapping peptides. Data are expressed as percentage of functional CD4 T_FH_ cells stimulated by vaccine immunogens. Tabulated data show mean +/- SEM.

A cytokine-independent method was used to identify and quantify antigen-specific germinal center (GC) T_FH_ cells (30) in each vaccine group to assess the contribution of T_FH_ for antigen-specific B cell survival and proliferation. T_FH_ responses specific for Env and Gag were measured in inguinal lymph nodes sampled after the second and third immunizations (**Figure 4**). Regardless of the vaccine strategy, the overall Env-specific T_FH_ responses were significantly higher than the Gag-specific T_FH_ responses (P < 0.0001). Overall Env-specific responses by NHP in the SAd7+Protein group were greater than Env responses in the DNA+Protein group (P = 0.0192), and they were also greater after two immunizations in the SAd7+Protein group (Week 6). However, Gag-specific responses were low and did not differ between the vaccine groups except after the third immunization (Week 14), when the DNA+Protein group had developed higher Gag responses compared to the SAd7+Protein group (P = 0.0124).

### Binding and Functional Antibody Responses

**Figure 5:**
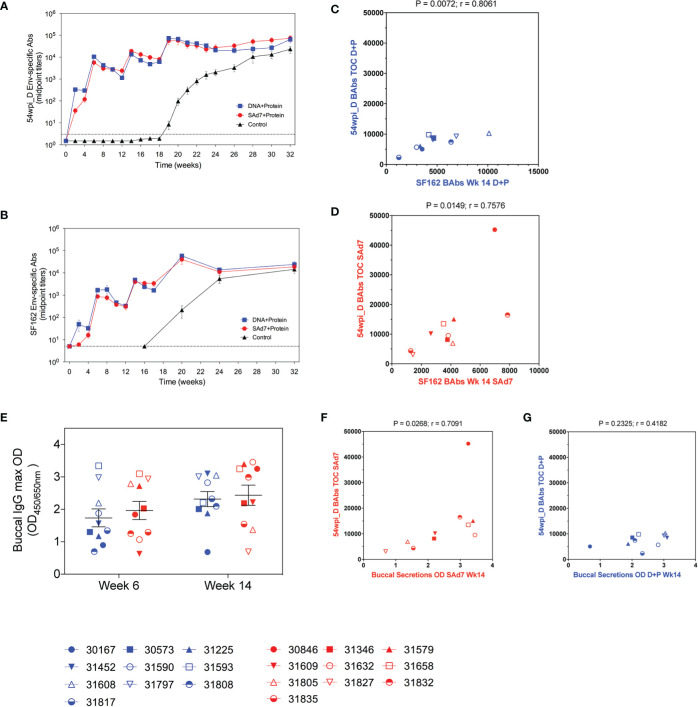
Longitudinal CAP257 54wpi_D-specific systemic and mucosal binding antibodies. Plasma samples from vaccinated macaques were tested longitudinally by ELISA for IgG binding antibodies to **(A)** autologous CAP257 54wpi_D and **(B)** heterologous HIV-1 SF162 gp140 trimeric proteins. Correlative analysis between the responses to autologous and heterologous Envs in the DNA+Protein group **(C)** and the SAd7+Protein group **(D)**. **(E)** Buccal secretions were tested for IgG content by ELISA against CAP257 54wpi_D gp140 protein after the second and third immunizations (Week 6 and Week 14, respectively) for IgG binding antibodies to CAP257 54wpi_D gp140 trimeric protein. Correlative analysis between the titers of immunogen-specific IgG in the plasma of animals at the time of challenge and IgG present in buccal secretions after the third immunization in the Sad7+Protein group **(F)** and the DNA+Protein group **(G)**. DNA+Protein group is in blue and SAd7+Protein group is in red, NHPs are identified by individual symbols.

Autologous antibody responses elicited by the two vaccine strategies were followed longitudinally by assessing binding antibody titers to CAP257 54wpi_D gp140 protein (**Figure 5A**). Env-binding antibodies were detected by the first vaccination in both vaccine groups and increased with subsequent immunizations. Overall, antibody titers measured in the plasma of macaques in both vaccine groups were comparable, and there was no antibody response in the Control group before the start of the challenge. As a comparison, heterologous antibody development to clade B, HIV-1 SF162 gp140 was also measured (**Figure 5B**). Plasma antibodies directed to the clade B Env were lower after the first two immunizations, but comparable to autologous antibody responses through subsequent immunizations, and the responses to both autologous and heterologous Envs are strongly correlative (**Figures 5C, D**). Rectal mucosal secretions were not collected during the immunizations or at the time of challenge to avoid interference with the integrity of the rectal lining before SHIV IR challenges. Buccal secretion samples harvested 2 weeks after the second and third immunizations were tested for the presence of CAP257 54wpi_D Env-specific IgG and IgA mucosal antibodies (**Figure 5E**). No IgA antibodies directed to Envelope were detected. Mucosal IgG titers were similar between groups and remained stable through the third immunization. When compared, the titers of immunogen-specific IgG in the plasma of animals in the SAd7 group at the time of challenge (Week 16) correlated with IgG present in buccal secretions after the third immunization (P = 0.0268), but this correlation was not mirrored in the DNA+Protein group (P = 0.2325) (**Figures 5F, G)**.

**Figure 6:**
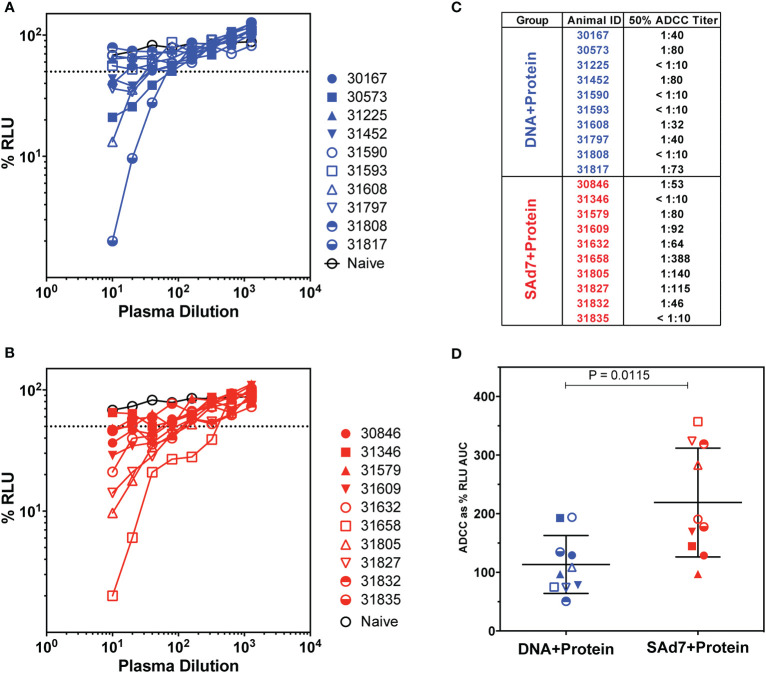
Antibody-dependent cellular cytotoxicity in macaque plasma. **(A, B)** Serially diluted plasma samples were tested for ADCC activity in a SHIV_SF162P3_ infected cell assay as described (15). ADCC responses were measured as the dose-dependent loss of luciferase activity in relative light units (RLU) after incubation in comparison to control wells containing NK cells and either infected (maximal) or uninfected (background) CEM.NKR-CCR5-sLTR-Luc cells in the absence of antibody. The dotted line indicates half-maximal lysis of infected cells. **(C)** 50% ADCC titers were calculated by linear regression. **(D)** %RLU values were used to determine area under the curve (AUC) values. Differences between log_10_-transformed %RLU values and 100% RLU, indicating no activity, were calculated at each plasma dilution tested. The sum of these differences for each sample was multiplied by the dilution factor.

Antibody-dependent cellular cytotoxicity (ADCC) was assessed in the plasma of vaccinated animals at the time of challenge (Week 16) (**Figure 6**) as previously described (15, 31). In order to measure killing of SHIV-infected cells, Natural Killer (NK) cell lines that have been transduced to express rhesus CD16 were used as effectors and target cells were a CD4+ T-cell line that expresses luciferase. Thus, the dose-dependent loss of luciferase activity was measured as an indication of antibody-mediated killing of virus-infected cells (**Figures 6A–B**). In DNA+Protein vaccinated macaques, six of 10 animals achieved at least 50% ADCC activity. Greater ADCC activity was seen in the SAd7+Protein group with eight of 10 animals generating at least 50% SHIV-infected cell killing activity in plasma (**Figure 6C**). Using Area Under the Curve (AUC) as the parameter to compare vaccine groups, the SAd7 vaccinated macaques generated greater ADCC activity in plasma compared to the DNA+Protein animals (P = 0.0115, **Figure 6D**).

**Figure 7:**
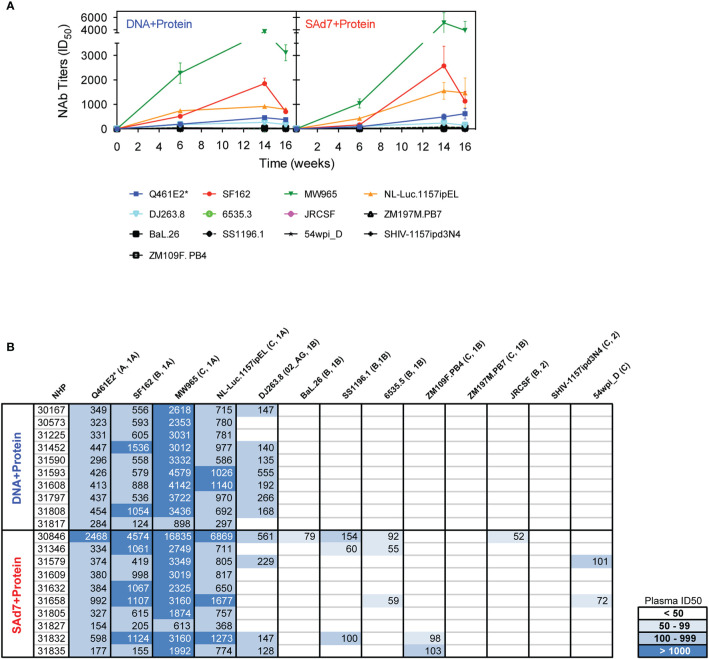
Heterologous and autologous neutralizing antibodies. **(A)** Longitudinal plasma samples from vaccinated macaques were tested for neutralization of 13 viruses, including the autologous CAP257 54wpi_D env pseudovirus, by TZM-bl assay 2 weeks after the second and third immunizations and at the time of challenge (Weeks 6, 14, and 16, respectively). **(B)** Neutralization titers of individual plasma samples of NHPs at time of challenge. Neutralization data are expressed as ID_50_, plasma dilution that neutralized 50% of the infecting virus, with the Clade and Tier information indicated in parentheses.

Neutralizing activity in plasma directed against 13 Tier 1 and Tier 2 pseudoviruses was followed longitudinally after the second and third immunizations (**Figure 7A**). NAb titers were detected after the second immunization in both vaccine groups against Tier 1 viruses, strongest against clade C, MW965. After the third immunization, titers peaked and were noticeably improved against clade C, NL4.Luc._1157ipEL_. NAb titers remained highest against Tier 1 viruses and are shown individually for each NHP at time of challenge (**Figure 7B**). Interestingly, several Tier 1B and Tier 2 viruses, including the Tier 2 autologous virus 54wpi_D, were neutralized by plasma from a few animals in the SAd7+Protein group and none in the DNA+Protein group, (**Table 2**). However, neither vaccine strategy elicited NAbs against the Tier 2 challenge virus SHIV-1157ipd3N4, which is an infectious molecular clone (32) (**Figure 7B**). Most relevant are the responses following the 3^rd^ immunization before to challenge.

**Table 2:** Mann-Whitney statistical analysis comparing neutralization responses elicited by the two vaccine strategies at time of challenge.

Virus	*P* value
Q461d1 (A)	0.9118
SF162 (B)	0.3150
MW965 (C)	0.3150
N-Luc._1157ipEL_ (C)	0.6842
DJ263.8 (AG)	0.4935
BaL.26 (B)	0.1603
SS1196.1 (B)	**0.0001**
6535.3 (B)	**0.0355**
ZM109.PB4 (C)	**0.0156**
ZM197.PB7 (C)	**0.0204**
JR-CSF (B)	**<0.0001**
SHIV-1157ipd3N4 (C)	>0.9999
54wpi_D (C)	**0.0251**

P values comparing the neutralization titers elicited by both vaccine strategies are indicated for each virus. Statistically significant P values are bolded.

Titers of neutralizing antibodies in the SAd7 group were greater compared to titers elicited in the DNA+Protein group against six of 13 viruses tested.

### Challenge Phase and SHIV in Blood and Tissues

**Figure 8:**
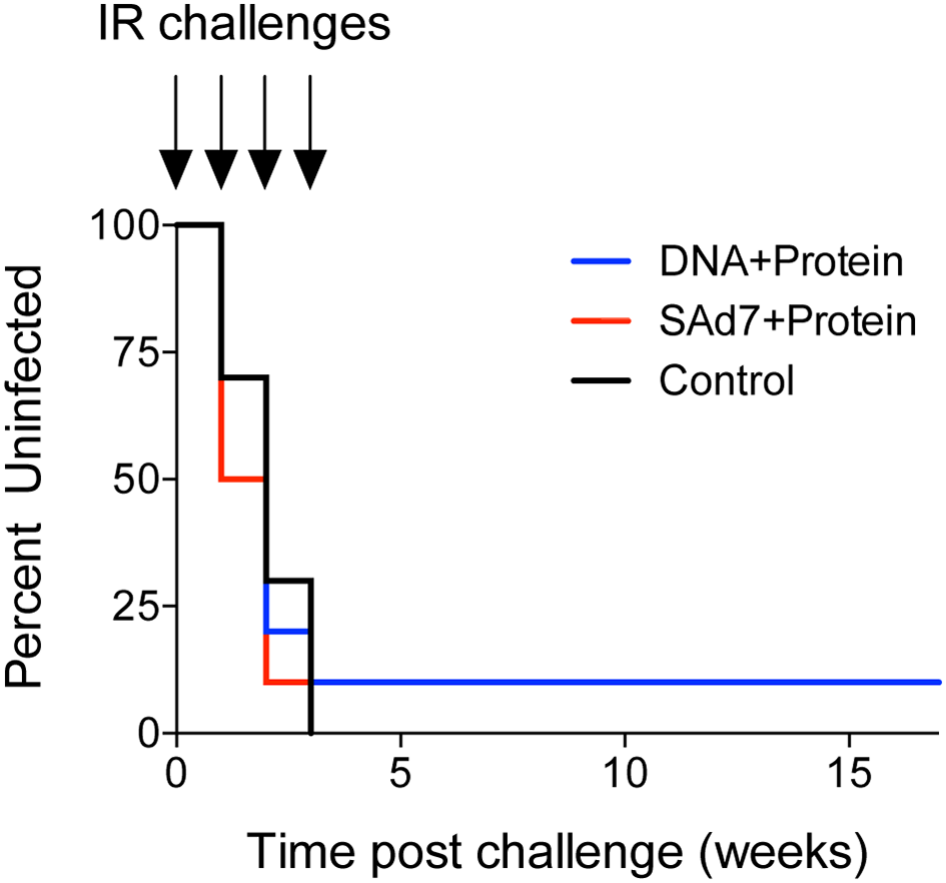
Onset of infection analysis. Modified Kaplan-Meier curve showing the % controllers in each experimental group over the observation period (time post challenge in weeks) based on plasma viral RNA loads.

**Figure 9:**
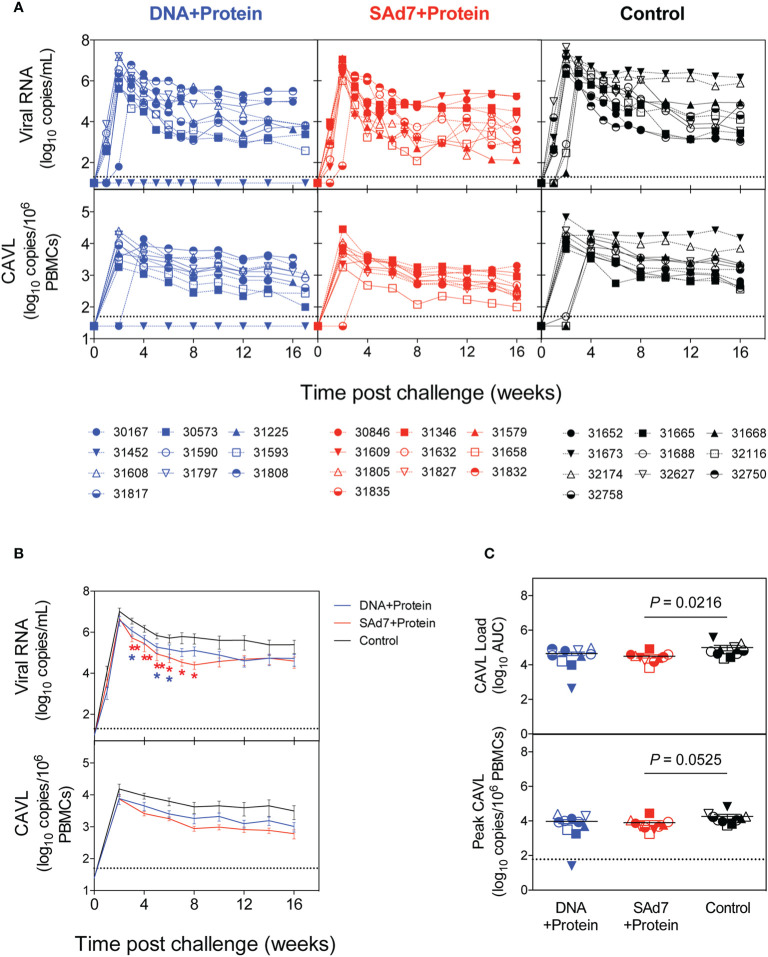
Plasma and cell-associated viral loads. **(A)** Individual longitudinal plasma and cell-associated viral RNA loads. Viral RNA loads (log_10_ RNA copies/ml) and viral DNA loads (log_10_ DNA copies/10^6^ PBMCs) were determined longitudinally by real-time RT-PCR. Individual animals are listed for each group with the DNA+Protein group in blue, SAd7+Protein group in red and Control group in black. **(B)** Group mean plasma and cell-associated viral RNA loads. Timepoints (weeks) indicated with an asterisk indicate a significant difference between either SAd7+ Protein (red) or DNA+Protein (blue) and controls PVL post challenge compared to Controls. P values for SAd7 are: **P=0.0024, **P=0.0014, **P=0.0025, *P=0.0188, *P=0.0301, *P=0.0126 for consecutive weeks (weeks 3–8). P values for DNA+Protein are: *P=0.0252, *P=0.0380, and *P=0.0450 for weeks 3, 5, and 6. **(C)** Cell-associated viral RNA loads and peak cell associated viral loads are reported as Area Under the Curve (AUC). P values are shown on each panel.

Macaques were challenged intrarectally (IR) each week with 5000 TCID_50_ units (by TZM-bl assay) of heterologous Tier 2 clade C SHIV-1157ipd3N4 virus until all control NHPs were infected. Four IR exposures to SHIV-1157ipd3N4 infected all controls and all SAd7+Protein vaccinated NHPs. In the DNA+Protein vaccine group, 9/10 animals became infected with NHP 31452 remaining aviremic. A modified Kaplan-Meier plot depicts the time to infection in each vaccine group and controls, and the rate of infection was comparable between vaccine groups (**Figure 8**, P = 0.4320). All macaques were followed longitudinally for up to 18 weeks for evaluation of plasma and cell-associated viral loads in individual macaques (**Figure 9A**) and as group averages (**Figure 9B**). Peak plasma virus levels in the vaccinated NHPs were not different from those in the Control animals, and no differences were found when measured longitudinally as area under the curve (AUC) (data not shown). However, an analysis of longitudinal plasma viral loads (PVL) in the weeks post challenge was used to compare PVL differences between groups overall and at each timepoint measured. The analysis revealed a highly significant overall effect (P < 0.0001) and further revealed that at each week (weeks 3–8), the SAd7+Protein group PVL was significantly lower than the Control group. The DNA+Protein group PVL differed from the Control group only at weeks 3, 5, and 6 post challenge. However, in SAd7+Protein vaccinated macaques, copies of viral DNA in PBMCs (CAVL, AUC) were significantly lower compared to control animals (P = 0.0216) and peak cell-associated DNA copies trended close to statistical significance (P = 0.0525) (**Figure 9C**).

**Figure 10:**
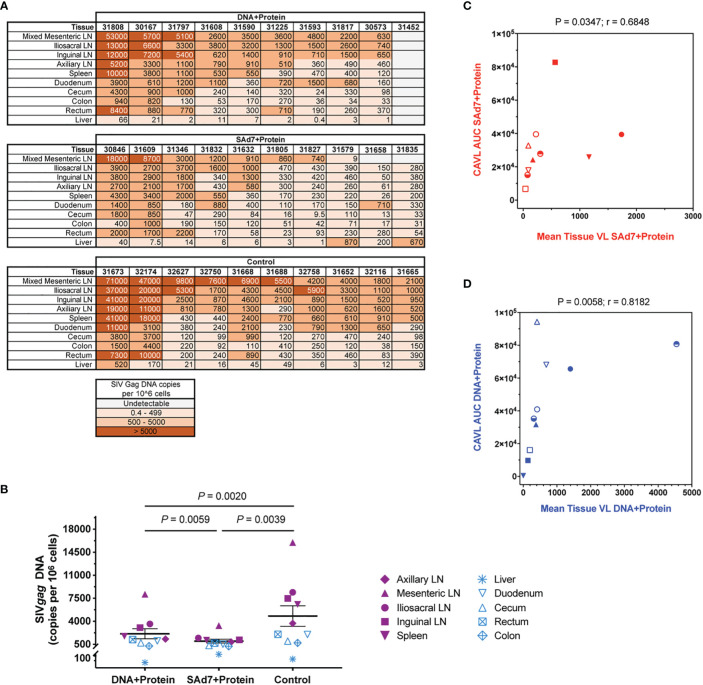
Tissue viral loads. Viral loads were determined in 10 tissues at time of necropsy. **(A)** Quantified SIV Gag DNA copies per 10^6^ cells in all animals are shown in heatmap layout and color key is shown. **(B)** Group comparison of tissue virus burden mean values. Individual tissues are listed and lymphoid tissues are identified by filled purple symbols while non-lymphoid tissues are identified by open blue symbols. P values are shown. **(C, D)** Correlative analysis of cell-associated virus and tissue virus burden in each group. Spearman correlation P and r values are shown. Group colors and animal symbols are the same as in **Figure 9**.

At necropsy, tissue viral DNA copies were evaluated in ten individual tissues from all 30 animals (**Figure 10A**). Greater numbers of viral copies in lymphoid tissues are seen in more animals in the Control group and in the DNA+Protein group compared with the SAd7+Protein group. This difference in lymphoid viral DNA is reflected in the overall difference of tissue DNA. The tissue viral copies were significantly greater in the Controls compared to the DNA+Protein group (P = 0.0020) and to the SAd7+Protein group (P = 0.0039). Also, a significant difference in tissue viral DNA copies was found between the two vaccine groups (P = 0.0059) (**Figure 10B**). Individual tissue types were also compared among the vaccinated animals and the control animals. Two types of lymphoid specimens showed differences between vaccinated and unvaccinated groups. The mesenteric lymph nodes of Control group animals had significantly higher viral copies compared to the SAd7+Protein vaccine group animals (P = 0.0498). In addition, viral DNA associated with the iliosacral lymph nodes in Controls were significantly higher compared to the same tissues of SAd7+Protein vaccinated animals (P = 0.0056). Finally, for both vaccine strategies, tissue viral DNA copies correlated with cell-associated viral copies in blood cells (**Figures 10C, D**).

## Discussion

In this macaque study, we compared the immunogenicity of HIV clade C env antigens delivered by replication-competent SAd7+Protein prime-boost vaccine *versus* a DNA+Protein co-administration vaccine strategy. The env antigens used in this study are derived from envs isolated from CAP257, a participant in the South African CAPRISA cohort who developed neutralization breadth within 18 months of HIV infection (5). The choice of these specific envs was based on DNA+Protein comparative immunogenicity studies in rabbits and macaques (6). In the immunogenicity studies, these envs induced T_FH_ responses along with strong Tier 1 and modest Tier 2 NAbs. The promising antibody and T_FH_ responses prompted us to evaluate them in a rhesus macaque mucosal viral challenge model where we could determine their protective efficacy against a Tier 2 SHIV. In addition, we were also interested in testing two different vaccine approaches by comparing head-to-head the delivery of the same Env antigens by SAd7 prime-Protein boost versus plasmid DNA+Protein co-immunization. The SAd7+Protein vaccine approach was recently tested using a different clade C Env (1086C) immunogen (12), and this vaccine resulted in some protection and reduction in tissue viral DNA following a Tier 1 clade C challenge.

Overall, both DNA+Protein and SAd7+Protein vaccine strategies elicited comparable antigen-specific cellular and humoral immune responses, but there were important differences especially regarding functional antibody responses which were stronger in the SAd7+Protein group. Both vaccine approaches elicited greater Env-specific cellular responses than Gag-specific responses, as we and others have also observed in previous studies using adenovirus vectors (12, 33). DNA primes are known to elicit strong T cell stimulation (34) but the measured ELISPOT T cell responses were not significantly higher in the DNA+Protein group compared to the SAd7+protein group and we hypothesize that the temporal co-delivery of the DNA and protein vaccine components may have helped induce a more balanced cellular and humoral stimulation than a DNA prime. Indeed, the SAd7+Protein prime-boost and the DNA+Protein coimmunization strategies produced similar levels of systemic and mucosal autologous IgG antibodies, although neither vaccine induced measurable mucosal Env-specific IgA. All vaccinated macaques had neutralizing antibodies against clades A, B, and C Tier 1A viruses. The SAd7+Protein regimen had consistently higher percentages of Env-specific T_FH_ cells in lymph nodes. Animals in this group also exhibited low NAbs against several Tier 1B viruses and a few animals had low level activity against the Tier 2 CAP257 54wpi_D autologous virus, but there was no evidence of neutralization of the Tier 2 challenge virus, SHIV-1157ipd3N4. Variation in the levels of Tier 2 autologous NAbs in macaques has been observed after vaccination with Env SOSIP, and the better responses were correlated with T_FH_ responses in germinal centers (30). Modest levels of ADCC activity were generated in several animals in each vaccine group, and ADCC responses were significantly greater at the time of challenge in the SAd7+protein group, which had 80% responders. We propose that the improved responses obtained in the SAd7+Protein group are due to the replication-competent priming vector that elicits a higher and/or more sustained stimulation of the immune system compared to the DNA+Protein strategy. Improvement of immune responses by replication-competent vectors have also been observed with poxvirus-based vectors (35).

Despite these humoral and cellular responses, neither vaccine regimen afforded significant protection against a rigorous heterologous repeated titered intrarectal challenge with Tier 2 clade C SHIV-1157ipd3N4 (20, 21). The infection rate was not different between vaccine groups and the plasma viral burden in the vaccinated NHPs was not significantly lower than in the control animals. These results contrast with our previous data (12) where time to infection with the Tier 1 clade C SHIV-1157ipEL-p was significantly longer in the SAd7 vaccine group than in the control group and where plasma viremia was significantly lower in infected vaccinated macaques than in controls, similar to results obtained with other adenovirus vectors (36). We propose that the lack of protection from acquisition is due in part to the relative potency of the SHIVs used, a Tier 2 SHIV in the current study versus a Tier 1 SHIV in our previous work (12). In the current study, all SAd7+Protein vaccinated NHPs became infected while one of DNA+Protein macaques (NHP 31452) remained aviremic by all tests performed. While ADCC activity has been associated with protection in the NHP challenge model (37) and correlated with reduction of risk of acquisition in the RV144 clinical trial (38), we found no correlation with ADCC and plasma or cell-associated viral loads in either group in this study. Failure to elicit protective immunity may also be due in part to the use of an unconstrained trimeric Env gp140 protein in both vaccines.

To date only one Env-based adenovirus-prime followed by Env protein-boost strategy reached a 50% protective efficacy in vaccinated NHPs against a heterologous Tier 2 SHIV (36). Based on virus infectious doses, our study was a stringent efficacy test with a dose ten times higher (500 *vs.* 5000 TCID_50_/ml) than the challenge dose in that study. Even though this Ad26 vaccine was immunogenic and protective in macaques, an attenuated version of this Ad was not immunogenic when recently assessed in humans *via* a different delivery route (39). Similar to our results, other vaccine strategies have not been successful in showing protection against heterologous Tier 2 SHIV mucosal challenges (21, 40, 41) but were able to provide enhanced viral control (42). Recently there has been some success in protection using a SOSIP protein vaccine followed by a homologous Tier 2 challenge virus (2), and protection not surprisingly correlated with the potency of autologous NAbs. The failure to obtain significant protection from heterologous SHIV infection with either of these vaccine strategies is likely due to a failure to elicit NAbs effective against the heterologous challenge virus.

When viral vaccines do not provide protection from infection, they might nonetheless offer some benefits if they could lower viremia or tissue associated virus in infected vaccinees. One such example is the rhCMV-based vaccine that generates potent T effector memory cells against SIV antigens and results in tight control of viremia in ~50% of macaques infected with SIVmac239 (43, 44), and there is evidence that these animals can go on to eliminate the virus (3). We obtained this type of outcome with our previous adenovirus-based vaccine (12), where the cell-associated viral loads were significantly lower in SAd7+Protein vaccinated macaques compared to control animals. In related studies in primates, we showed that bNAbs can limit viral seeding when present within 24 to 30 h of exposure to Tier 2 SHIV_SF162P3_ (28, 45) and that it is possible to reduce the levels of tissue viral DNA in macaques exposed to Tier 1 SHIV in the presence of a single human monoclonal antibody directed to V1V2 (46). In the current experiment, both regimens significantly lowered viral DNA copies in lymphoid and gut tissues tested. In addition, at time of necropsy, the SAd7+Protein group had a significantly lower number of viral copies in mesenteric and iliosacral lymph nodes compared to the Control group. We hypothesize that the combined humoral and cellular responses induced by SAd7 priming could have contributed to reducing the viral burden in infected animals. There was no single immunological correlate for tissue viral DNA reduction identified in this study, but the study was powered sufficiently to discriminate between the vaccine arms. Both ADCC and neutralizing antibodies were more prevalent and potent in the SAd7+Protein group, which had the lowest viral DNA in tissues; thus, further emphasizing the priming role of replication-competent SAd7 vector. Because viral DNA copies are strongly correlated with plasma viral DNA in other Tier 2 SHIV studies (10), we posit that reduction in tissue viral DNA is a benchmark for vaccines.

In summary, our data demonstrate that Ad prime-protein boost and DNA+Protein co-immunization strategies using clade C Env-based immunogens generated similar levels of cellular immunity, but the SAd7+protein approach induced Env-specific T_FH_ cells more rapidly, more potent ADCC responses, more evidence of heterologous NAbs, and more pronounced effect upon tissue viral seeding. This study highlights the difficulty in designing a protective vaccine against a heterologous mucosal Tier 2 challenge. However, since T/F viruses are mainly categorized as Tier 2 (47, 48), defeating such a challenge is paramount for the development of an efficacious HIV vaccine and overcoming this obstacle remains the main goal for which HIV vaccine researchers strive. Nonetheless, reduction of virus in tissues by vaccine-induced immunity is an encouraging step.

## Data Availability Statement

The raw data supporting the conclusions of this article will be made available by the authors, without undue reservation.

## Ethics Statement

The animal study was reviewed and approved by OHSU West Campus Institutional Animal Care and Use Committee.

## Author Contributions

DM selected and designed the Env vaccine constructs, conceived the study, analyzed experiments, prepared figures and wrote the manuscript. LV and JM designed and produced the SAd7 vaccine construct. PB and BK performed ELISA and neutralization assays. DS performed the ADCC assays. JR performed the ELISPOT and T_FH_ assays. DNS produced the gp140 proteins. SP produced the gp160 vaccines. CW isolated envelope clones. HR motif-optimized the vaccine sequences. DF provided the PMED XR-1 gene gun device. BP performed statistical analyses. SL produced the challenge virus. MA supervised the animal study. JW, RMR, JS and PM assisted with data interpretation and manuscript preparation. AH analyzed data, prepared figures and assisted with manuscript preparation. JA supervised the production of the SAd7 vaccine, contributed to the design of the study, and assisted with data interpretation and manuscript preparation. NH secured funding, conceived the study, assisted with data analysis and interpretation and wrote the manuscript. All authors contributed to the article and approved the submitted version.

## Funding

Research funding was provided by HHS grants from the National Institutes of Health, R44 AI091546-03 (JA, NH), P01 AI078064 (NH), U42 OD023038 (MA), and P51 OD011092 (NH, JS, MA).

## Conflict of Interest

JM and LV are current paid employees of Emergent BioSolutions and also own Emergent BioSolutions shares. JA is currently a paid consultant of Emergent BioSolutions. JA is listed as an author on a pending U.S. patent application No. 12/847, 767. JW is an advisor to REGENXBIO, Dimension Therapeutics, and Solid Gene Therapy, and is a founder of, holds equity in, and has a sponsored research agreement with REGENXBIO and Dimension Therapeutics. In addition, he is a consultant to several biopharmaceutical companies and is an inventor on patents licensed to various biopharmaceutical companies.

The remaining authors declare that the research was conducted in the absence of any commercial or financial relationships that could be construed as a potential conflict of interest.
